# A novel variant leads to WT1-related nephrotic syndrome and differences of sex development: a case report

**DOI:** 10.3389/fped.2025.1657533

**Published:** 2025-09-04

**Authors:** Shan Gao, Dahai Wang, Xingmei Ding, Cui Bai, Nana Nie, Hong Chang, Ranran Zhang, Jia Liu, Qiuye Zhang, Lin Liu, Yi Lin

**Affiliations:** ^1^ Department of Pediatric Nephrology, Rheumatology and Immunology, The Affiliated Hospital of Qingdao University, Qingdao, China; ^2^ Department of Pediatrics, Qingdao West Coast New Area District Hospital, Qingdao, China

**Keywords:** *WT1* gene, Frasier syndrome, nephrotic syndrome, focal segmental glomerulosclerosis, minigene experiment

## Abstract

**Background:**

*The Wilms Tumour gene 1* (*WT1*, NM_024426.6) holds significant importance in the developmental processes of the kidneys and gonads. Herein, we report a case of nephrotic syndrome and differences of sex development in a patient with novel mutations in *WT1* gene.

**Methods:**

The child, identified as female based on social gender, exhibited symptoms at 6 years of age and was diagnosed with steroid-resistant nephrotic syndrome (SRNS). Renal biopsy findings indicated focal segmental glomerulosclerosis. Whole-exome sequencing unveiled a novel variant, c.1447 + 6(IVS9)T > C, in the *WT1* gene, and karyotypic analysis revealed 46, XY, aligning with the phenotypic presentation of Frasier syndrome (FS, OMIM#136680) associated with *WT1* gene mutation. The influence of gene variants on mRNA splicing was examined using *in vitro* minigene assays.

**Results:**

The variant was classified as likely pathogenic (PS2 + PM2_Supporting + PP3) in accordance with American College of Medical Genetics and Genomics (ACMG) guidelines. *in vitro* minigene experiments demonstrated that the c.1447 + 6(IVS9)T > C variant altered the splicing pattern of exon 9 in the *WT1* gene from two isoforms to a single form, thereby supporting its pathogenicity.

**Conclusion:**

Through high-throughput sequencing and *in vitro* minigene splicing experiments, the c.1447 + 6T > C variant in the *WT1* gene was supported as the underlying genetic cause in the child patient, thereby expanding the spectrum of gene variants of *WT1 gene* and enhancing our comprehension of the molecular pathogenesis of this disorder.

## 1 Introduction

*The Wilms Tumour gene 1* (*WT1*), initially identified in 1990 among patients with Wilms’ tumour, encodes a transcription factor characterized by a zinc-finger-like structure. This gene holds significant importance in the developmental processes of the kidneys and gonads and has been implicated in a range of disorders, including Denys-Drash syndrome (OMIM#194080), FS, Wilms' tumour (OMIM#194070), Meacham syndrome(OMIM#608978), and WAGR syndrome(OMIM#194072) (1, 2). Notably, all these conditions are inherited in an autosomal dominant pattern.

FS is characterized predominantly by 46, XY gonadal dysgenesis. Nephropathy typically emerges between the ages of 2–6 years, often serving as the initial clinical manifestation. Patients exhibit resistance to steroid treatment, with the condition progressively advancing to end-stage renal disease. Renal histopathology predominantly features focal segmental glomerulosclerosis (FSGS) (3). This study presents the clinical data of a rare pediatric case of FS, with the aim of deepening clinicians' understanding of this syndrome. Furthermore, as this variant site has not been previously documented, it contributes to expanding the spectrum of variations in the *WT1* gene.

## 2 Case report

**Figure 1:**
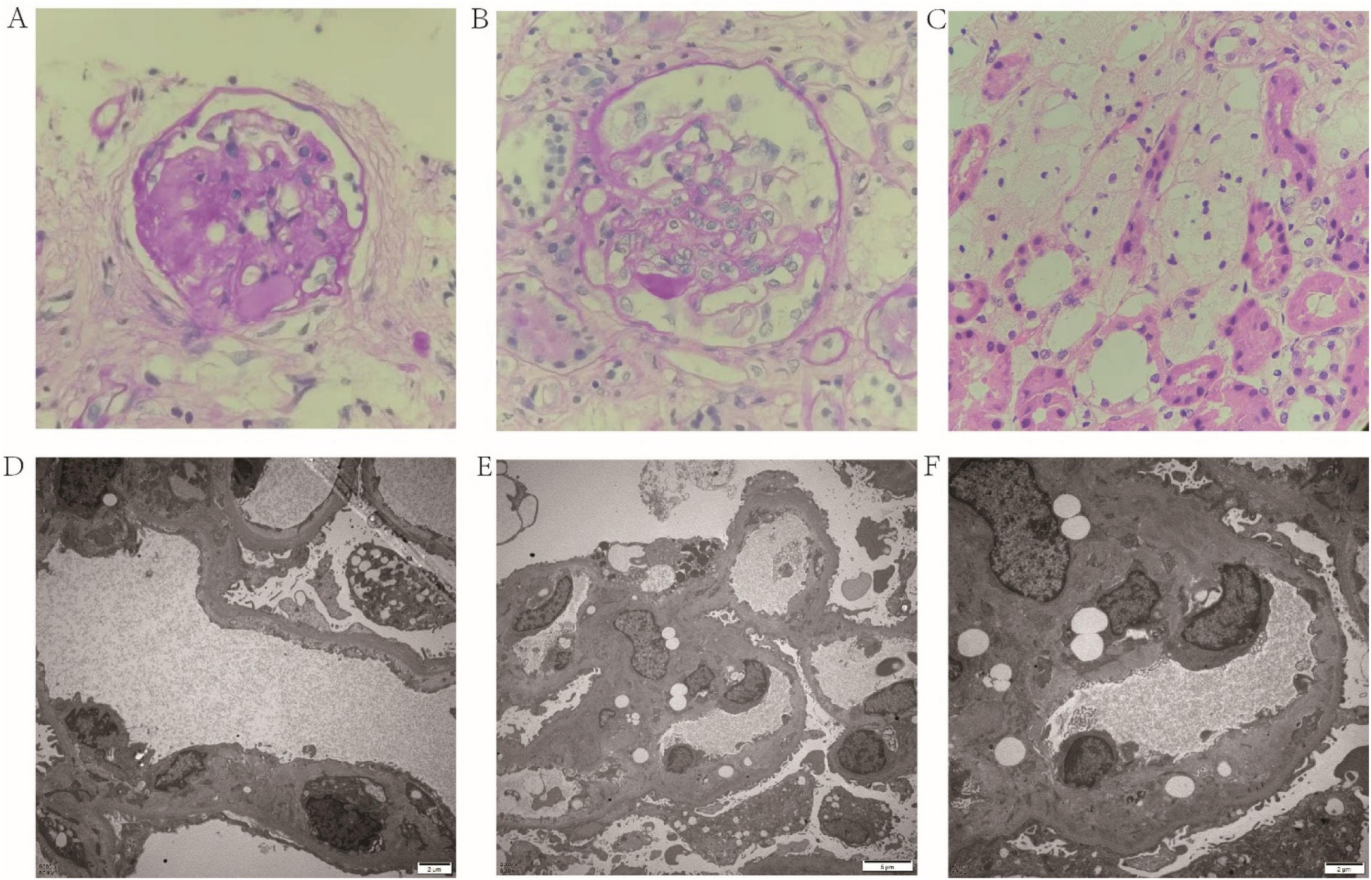
The pathological characteristics of kidney sections. Light micrograph **(A–C)**: Two segmental glomerulosclerosis (**A,B** HE × 400), mild acute lesions with chronic changes in the renal tubular interstitium (**C**, HE × 400). Electron Microscopy **(D–F)**: Widening of partial subendothelial spaces in the glomerulus accompanied by double-contour sign (**D**, ×5,000; **E**, ×3,000; **F**, ×6,000), Figure 1F is a magnified subregion od Figure 1E. HE, hematoxylin and eosin.

A 6-year-10-month-old female child presented to the Department of Pediatric Nephrology, Rheumatology and Immunology of the Affiliated Hospital of Qingdao University in March 2024 with complaints of “bilateral eyelid swelling for 8 days, exacerbated over the past 4 days”. Four days prior to admission, she had been diagnosed with NS at an external hospital, marked by hypoalbuminemia (Albumin 17.1 g/L), hyperlipidemia (total cholesterol 15.78 mmol/L), and significant proteinuria (urinary albumin ++++), in the absence of hematuria. Renal function and serum complements levels were within normal limits, and an ultrasound of the urinary system showed no abnormalities. She underwent a standard 4-week course of corticosteroid therapy (including two rounds of pulse steroid therapy), but follow-up urinalysis revealed persistent proteinuria (+++), indicating SRNS. Consequently, tacrolimus was added to her treatment regimen. A renal biopsy was performed, and light microscopy identified segmental sclerosis in 2 out of 24 glomeruli and one globally sclerosed glomerulus, suggesting FSGS. Additionally, mild acute tubulointerstitial changes with signs of chronicity and the accumulation of numerous foam cells were observed (Figures 1A–C). Electron microscopy revealed widening of the subendothelial space in some glomeruli with double-contour formation, segmental worm-eaten alterations, and scattered electron-dense deposits in the mesangial regions and subepithelial spaces of individual glomeruli, indicative of thrombotic microangiopathy (TMA)-like lesions (Figures 1D–F).

**Figure 2:**
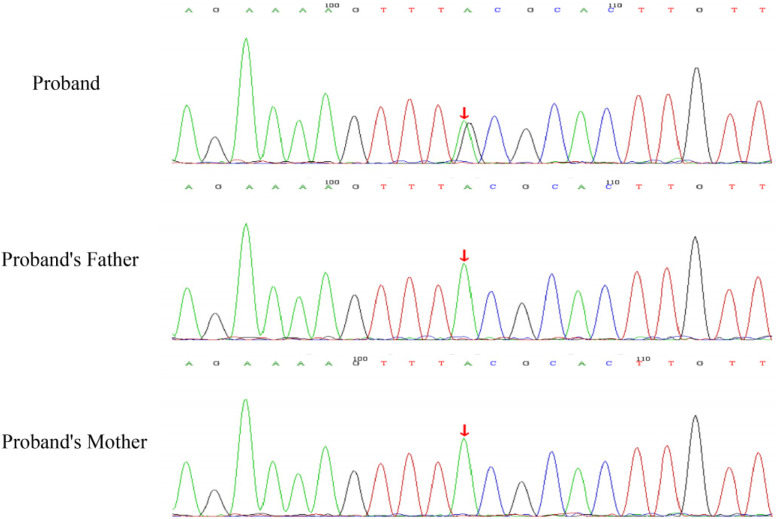
Sanger sequencing validation findings for the WT1 gene in the child and their parents: the child harbors a heterozygous c.1447 + 6T > C variant, whereas both parents display the wild-type sequence at this specific locus (the variant site is denoted by an arrow).

After obtaining consent from the pediatric patient's guardians, 2 ml peripheral blood samples were collected from the patient and each of her parents, anticoagulated with EDTA, for whole-exome sequencing. Dual-end sequencing was performed using the Illumina HiSeq X ten high-throughput sequencing platform. After sequencing, genes that lacked sufficient evidence of pathogenicity were excluded as candidate genes. The pathogenicity of the variant site was analyzed according to the 2015 guidelines of the American College of Medical Genetics and Genomics (ACMG). Exome sequencing unveiled a variant, c.1447 + 6T > C splicing variant was identified in the 9th intron of the patient's WT1 gene on chromosome 11, which was confirmed as a *de novo* variant through Sanger sequencing. Neither parent carried this variant (Figure 2). Sanger sequencing verified that neither parent carried this variant, indicating it was a *de novo* variant, which serves as strong pathogenic evidence (PS2). The frequency of this variant in normal population databases was negligible, thus classifying it as a low-frequency variant (PM2). The spliceAI prediction score was 0.70, suggesting an impact on splicing. Based on the relevant ACMG guidelines, this variant was judged as a suspected pathogenic variant, with no relevant reports of this site in the literature database.

To further validate the pathogenicity of this intronic splicing variant, we conducted an *in vitro* minigene splicing assay. Using the pMini-CopGFP plasmid as the vector, we amplified two fragments (WT1-A and WT1-B) from the wild-type WT1 gene using corresponding primers and constructed wild-type WT1-A and WT1-B plasmids. Mutant primers were designed based on these wild-type plasmids to obtain the WT1-MT fragment, and a mutant WT1-MT plasmid was constructed. The sequences of the primers used are listed in Table 1. The constructed plasmids were transfected into 293T cells, and reverse transcription primers (see Table 1) were designed for subsequent RT-PCR. The transcription products were electrophoresed on an agarose gel to observe changes in the lengths of the WT1-WT and WT1-MT fragments. The products were then analyzed by Sanger sequencing.

**Table 1:** The primers used in amplification of target fragments and reverse transcription-polymerase chain reaction (RT-PCR).

Primer Name	Upstream primer	Downstream primer	Usage
WT1-A	TAAGCTTGGTACCGAGCTCGGATCCGTGAGAAACCATACCAGTGTGACTTCA	TCTACCCTGGGATCACAGCTCACTGCAGCCTCAGCCT	Amplify target gene fragment
WT1-B	AGCTGTGATCCCAGGGTAGATGACCCAGAGGCTTCT	TTAAACGGGCCCTCTAGACTCGAGTCATGTTTCTCTGATGCATGTTGTGAT	Amplify target gene fragment
WT1-MT	GGTAAAACAAGTGCGcAAACTTTTCTTCACATTTATTTTTCATTATT	TgCGCACTTGTTTTACCTGTATGAGTCCTGGT	Point mutation in mutant plasmid
E8-E10	GGCTAACTAGAGAACCCACTGCTTA	TCATGTTTCTCTGATGCATGTTGTG	RT -PCR primers

**Figure 3:**
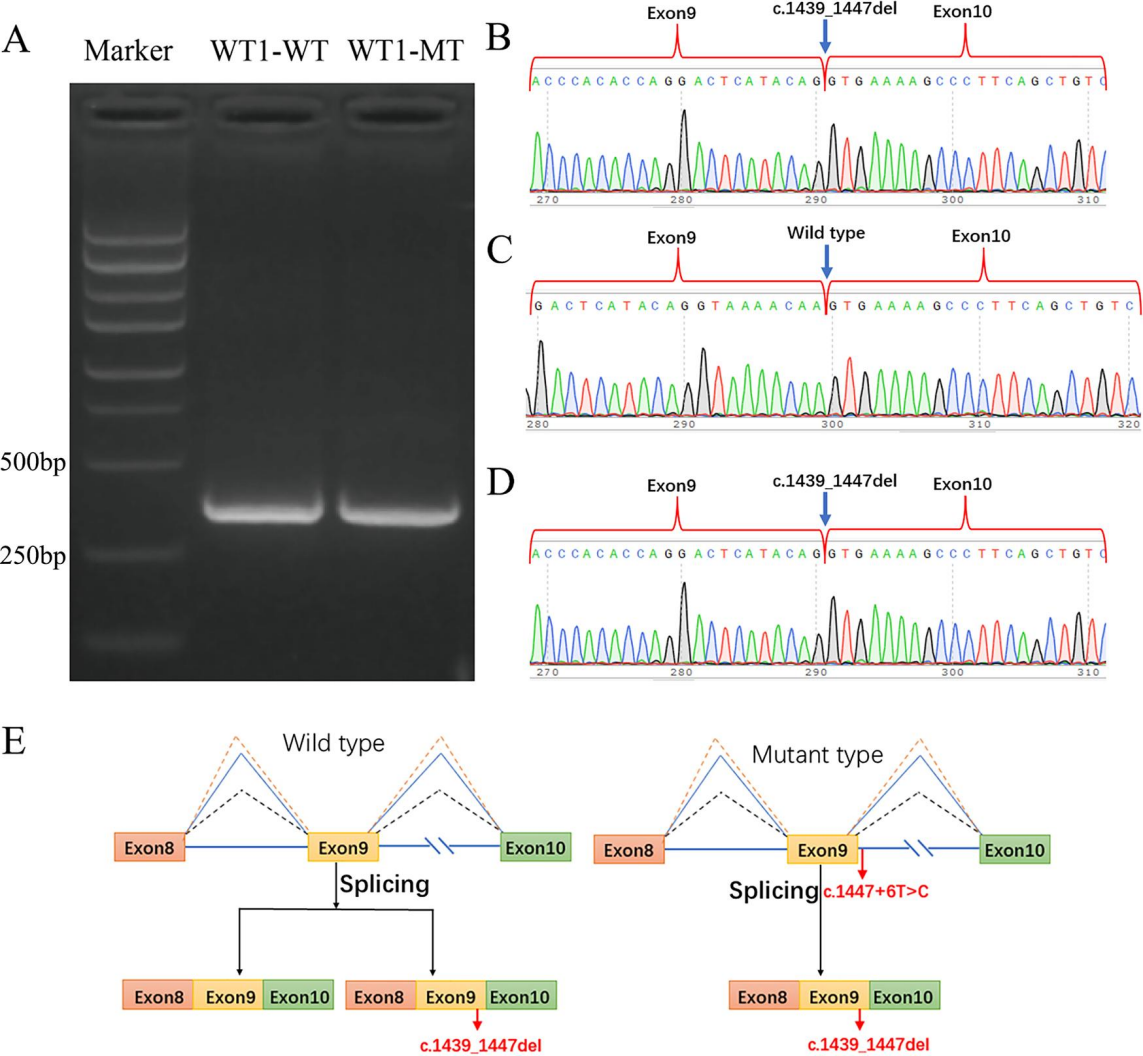
**(A)** Western blot of PT-PCR transcript products from the minigene experiment: both WT1-WT and WT1-MT transcripts aligned with the anticipated band length. Sanger sequencing of PT-PCR transcript products: **(B,C)** Two splicing isoforms of WT1-WT. **(D)** One splicing of WT1-MT, and the transcript products of **B** and **D** are identical. **(E)** Minigene splicing functional analysis of the variant c.1447 + 6(IVS9)T > C demonstrates that the WT allele displays two splicing isoforms, whereas the MT allele exhibits only one splicing isoform.

PCR amplification of the normal group generated an expected product size of 353 bp on an agarose. Both WT1-WT and WT1-MT transcripts aligned with the anticipated band length, could not indicate whether there was a splicing change in the mutant plasmid (Figure 3A). Sanger sequencing identified two splicing isoforms in WT1-WT: one retaining full exons 8, 9, and 10 (353 bp) (Figure 3C), and the other lacking a 9 bp sequence (c.1439_1447del, p.481_483delLysThrSer) in exon 9 (Figure 3B). In contrast, WT1-MT exclusively exhibited the c.1439_1447del splicing variant (Figure 3D). Thus, the *WT1* gene variant c.1447 + 6T > C shifted splicing from two isoforms to one. This variant is a hemizygous *de novo* variant, consistent with an autosomal dominant inheritance pattern, and the patient's clinical presentation aligns with *WT1* gene variant-associated disorders. Collectively, these findings classify the c.1447 + 6T > C splicing variant in *WT1* as pathogenic.

We performed a chromosomal karyotype analysis on the patient, which revealed a 46, XY karyotype. A gynecological ultrasound demonstrated a left ovary measuring 0.6 × 0.2 cm with no abnormal internal echoes and a suspected right ovary measuring 0.6 × 0.2 cm with no discernible follicular echoes. Given the patient's female gender identity, diagnosis of SRNS at age 6, renal pathology consistent with FSGS, and a exome sequencing result revealing a *WT1* gene variant, the minigene *in vitro* splicing assay supported this variant as pathogenic. Consequently, we supported the diagnosis of FSGS caused by a *WT1* gene variation. Currently, the patient has been tapered off prednisone (total duration: 3.5 months) and treated with tacrolimus. The patient presents with urine protein levels of +++ to ++++, no significant edema, preserved renal function, and no evidence of renal or gonadal neoplasms.

## 3 Discussion

FS is a clinical condition characterized by progressive nephropathy, male pseudohermaphroditism, and a high incidence of gonadal tumors. It was initially reported by Frasier in 1964 (4), and in 1997, Barbaux et al. identified *WT1* gene variants in patients affected by this syndrome (5). The *WT1* gene is located on chromosome 11p13, comprises 10 exons, and encodes a 517-amino-acid zinc-finger-like transcription factor (6). Exons 1–6 of the *WT1* gene encode the transcriptional regulatory domain of the WT1 protein, whereas exons 7–10 encode the four zinc-finger motifs responsible for binding to DNA or RNA, respectively (7).

The *WT1* gene contains alternative splicing sites within exons 5 and 9, leading to the generation of four isoforms of the WT1 protein: WT1 + 17aa/−17aa and WT1 + KTS/−KTS. Studies have confirmed that these isoforms exhibit distinct nuclear localization patterns, DNA-binding domains, and transcriptional activities, enabling them to regulate the differentiation processes of various cell types. Specifically, WT1 + KTS is crucial for maintaining the normal function of glomerular podocytes, whereas WT1−KTS is essential for the development of embryonic gonads. To ensure the proper development of both the kidneys and gonads, the normal ratio between these two isoforms should be approximately 3:2 (8).

More than ten *WT1* gene variants associated with FS have been documented, predominantly involving splicing variants in intron 9. Notable hotspot variants include c.1447 + 4C > T and c.1447 + 5G > A (8). These splicing variants, primarily located at the 5′ end adjacent to the 3′ end of exon 9, can disrupt the normal ratio of WT1 + KTS to WT1-KTS isoforms, shifting it from 3:2 to 1:3.2–1:3.5. This imbalance inhibits the development of male sexual organs, resulting in gonadal dysgenesis manifestations such as ambiguous genitalia, hypospadias, cryptorchidism, or even complete female external genitalia in 46, XY males. The c.1447 + 6T > C variant site in this pediatric patient has not been reported previously. The minigene experiment supported that the variant at this site could lead to a change in the splicing pattern of exon 9 from two forms to one (only expressing the KTS− isoforms), thereby predicting an increase in the ratio of KTS− to KTS+ isoforms, which may result in developmental abnormalities of the gonads and kidneys. This result is consistent with the research findings of Smith C (9). This result differs from the previously reported ratio of 1:3, possibly due to the fact that these experiments only detect the levels of variant mRNA *in vitro* and cannot truly reflect the expression levels of alleles *in vivo*. Therefore, there is a difference between the two results.

Variants in the *WT1* gene are linked to an elevated risk of developing Wilms’ tumor and gonadoblastoma, with FS carrying a moderate risk profile of 5%–20%. A study analyzing 126 patients with *WT1* intron 9 variants identified a Wilms' tumor prevalence of 3% (8). Gonadoblastoma predominantly manifests in the dysgenic gonads of individuals with gonadal dysgenesis (10). Kollios reported a case involving a 6-year-old patient with FS who underwent prophylactic bilateral gonadectomy; although preoperative examinations did not detect gonadal tumors, postoperative pathology confirmed bilateral gonadoblastoma, underscoring the necessity for early gonadectomy in 46, XY patients with FS (11). Sex hormone replacement therapy should be initiated at an appropriate developmental stage to facilitate the maturation of secondary sexual characteristics. Regarding reproductive options, patients may consider utilizing donated oocytes and *in vitro* fertilization to achieve pregnancy.

Currently, there are no established standardized treatment protocols for FS within the fields of nephrology or oncology. For the management of NS, cyclosporine has been proposed as a therapeutic option, albeit with the caveat of an elevated risk of malignancy (12–14). In pediatric patients who progress to end-stage renal disease, renal transplantation is advised. A retrospective analysis has demonstrated that steroidresistant syndromes linked to pathogenic gene variants, such as *WT1*, do not exhibit recurrence following renal transplantation (15).

## 4 Conclusion

For pediatric patients diagnosed with SRNS, it is imperative to meticulously perform comprehensive pathological and genetic assessments. Given the limited documentation of gene variant sites, the identification of novel gene loci has significantly expanded the known spectrum of *WT1* gene variations, thereby offering invaluable insights into the pathogenesis of this condition.

## Data Availability

The original contributions presented in the study are included in the article/Supplementary Material, further inquiries can be directed to the corresponding author.

## References

[1] Torres-Cano A Portella-Fortuny R Müller-Sánchez C Porras-Marfil S Ramiro-Pareta M Chau Y-Y Deletion of Wt1 during early gonadogenesis leads to differences of sex development in male and female adult mice. PLoS Genet. (2022) 18:e1010240. 10.1371/journal.pgen.1010240 35704566 PMC9200307

[2] Sun P Wang J Ilyasova T Shumadalova A Agaverdiev M Wang C. The function of miRNAs in the process of kidney development. Noncoding RNA Res. (2023) 8:593–601. 10.1016/j.ncrna.2023.08.009 37680850 PMC10480480

[3] Huang Y-C Tsai M-C Tsai C-R Fu L-S. Frasier syndrome: a rare cause of refractory steroid-resistant nephrotic syndrome. Children. (2021) 8(8):617. 10.3390/children8080617 34438508 PMC8394468

[4] Frasier SD Bashore RA Mosier HD. Gonadoblastoma associated with pure gonadal dysgenesis in monozygous twins. J Pediatr. (1964) 64:740–5. 10.1016/S0022-3476(64)80622-3 14149008

[5] Barbaux S Niaudet P Gubler MC Grünfeld JP Jaubert F Kuttenn F Donor splice-site mutations in WT1 are responsible for frasier syndrome. Nat Genet. (1997) 17:467–70. 10.1038/ng1297-467 9398852

[6] Bielińska E Matiakowska K Haus O. Heterogeneity of human WT1 gene. Postepy Hig Med Dosw (Online). (2017) 71:595–601. 10.5604/01.3001.0010.3840 28791954

[7] Torban E Goodyer P. Wilms’ tumor gene 1: lessons from the interface between kidney development and cancer. Am J Physiol Renal Physiol. (2024) 326:F3–F19. 10.1152/ajprenal.00248.2023 37916284

[8] Tsuji Y Yamamura T Nagano C Horinouchi T Sakakibara N Ishiko S Systematic review of genotype-phenotype correlations in frasier syndrome. Kidney Int Rep. (2021) 6:2585–93. 10.1016/j.ekir.2021.07.010 34622098 PMC8484119

[9] Smith C Burugula BB Dunn I Aradhya S Kitzman JO Yee JL. High-throughput splicing assays identify known and 229 novel WT1 exon 9 variants in nephrotic syndrome. Kidney Int Rep. (2023) 8(10):2117–25. 10.1016/j.ekir.2023.07.033 37850022 PMC10577367

[10] Roth LM Cheng L. Gonadoblastoma: origin and outcome. Hum Pathol. (2020) 100:47–53. 10.1016/j.humpath.2019.11.005 31805291

[11] Kollios K Karipiadou A Papagianni M Traeger-Synodinos J Kosta K Savvidou P Bilateral gonadoblastoma in a 6-year-old girl with frasier syndrome: need for early preventive gonadectomy. J Pediatr Hematol Oncol. (2022) 44:471–3. 10.1097/MPH.0000000000002501 35700406

[12] Chiba Y Inoue CN. Once-daily low-dose cyclosporine A treatment with angiotensin blockade for long-term remission of nephropathy in frasier syndrome. Tohoku J Exp Med. (2019) 247:35–40. 10.1620/tjem.247.35 30651406

[13] Faul C Donnelly M Merscher-Gomez S Chang YH Franz S Delfgaauw J The actin cytoskeleton of kidney podocytes is a direct target of the antiproteinuric effect of cyclosporine A. Nat Med. (2008) 14:931–8. 10.1038/nm.1857 18724379 PMC4109287

[14] Sinha A Sharma S Gulati A Sharma A Agarwala S Hari P Frasier syndrome: early gonadoblastoma and cyclosporine responsiveness. Pediatr Nephrol. (2010) 25:2171–4. 10.1007/s00467-010-1518-x 20419325

[15] Morello W Proverbio E Puccio G Montini G. A systematic review and meta-analysis of the rate and risk factors for post-transplant disease recurrence in children with steroid resistant nephrotic syndrome. Kidney Int Rep. (2023) 8:254–64. 10.1016/j.ekir.2022.10.030 36815113 PMC9939313

